# Phylodynamic Analysis of Ebola Virus in the 2014 Sierra Leone Epidemic

**DOI:** 10.1371/currents.outbreaks.6f7025f1271821d4c815385b08f5f80e

**Published:** 2014-10-24

**Authors:** Erik Volz, Sergei Pond

**Affiliations:** Imperial College London; University of California San Diego

**Keywords:** ebola, ebolavirus, infectious disease, phylodynamics, superspreading

## Abstract

Background: The Ebola virus (EBOV) epidemic in Western Africa is the largest in recorded history and control efforts have so far failed to stem the rapid growth in the number of infections. Mathematical models serve a key role in estimating epidemic growth rates and the reproduction number (R0) from surveillance data and, recently, molecular sequence data. Phylodynamic analysis of existing EBOV time-stamped sequence data may provide independent estimates of the unobserved number of infections, reveal recent epidemiological history, and provide insight into selective pressures acting upon viral genes. Methods: We fit a series mathematical models of infectious disease dynamics to phylogenies estimated from 78 whole EBOV genomes collected from distinct patients in May and June of 2014 in Sierra Leone, and perform evolutionary analysis on these genomes combined with closely related EBOV genomes from previous outbreaks. Two analyses are conducted with values of the latent period that have been used in recent modelling efforts. We also examined the EBOV sequences for evidence of possible episodic adaptive molecular evolution during the 2014 outbreak. Results: We find evidence for adaptive evolution affecting L and GP protein coding regions of the EBOV genome, which is unlikely to bias molecular clock and phylodynamic analyses. We estimate R0=2.40 (95% HPD:1.54-3.87 ) if the mean latent period is 5.3 days, and R0=3.81, (95% HPD:2.47-6.3) if the mean latent period is 12.7 days. The estimated coefficient of variation (CV) of the number of transmissions per infected host is very high, and a large proportion of infections yield no transmissions. Conclusions: Estimates of R0 are sensitive to the unknown latent infectious period which can not be reliably estimated from genetic data alone. EBOV phylogenies show significant evidence for superspreading and extreme variance in the number of transmissions per infected individual during the early epidemic in Sierra Leone.

## Introduction

The 2014 Ebola virus in Western Africa is the largest Ebola epidemic in history and the number of infections continues to grow exponentially. The unprecedented rate of growth has negatively impacted the quality of epidemiological surveillance and has made it difficult to map and characterize the spread of the epidemic 13 . As local health and surveillance systems are overwhelmed, an unknown proportion of cases are unreported, are not isolated, and do not receive adequate treatment. Predictive modelling and evaluation of intervention efforts is also hampered by the rapid rate of increase and imperfect case reporting.

In the absence of complete surveillance data and contact tracing, mathematical models^20^ have provided valuable insights into the rate of epidemic growth and the reproduction number ( \begin{equation*}\small{R_0}\end{equation*} ). The reproduction number is a useful parameter for characterizing the difficulty of eradication. Early analyses based on case reporting by the World Health Organization (WHO) indicated that \begin{equation*}\small{ R_0}\end{equation*} differed substantially between countries^19^^,^ 17 . In some instances, estimates of \begin{equation*}\small{R_0}\end{equation*} based on different models are not in agreement, implying that they are sensitive to the assumptions of the mathematical framework used, and the exact data sets used for parameter estimation. Althaus^19^ estimated \begin{equation*}\small{ R_0}=2.53\end{equation*} (2.41-2.67) for Sierra Leone based on WHO case reports through late August 2014, whereas Fisman et al. 17 estimated \begin{equation*}\small{ R_0}=8.3\end{equation*} for Sierra Leone using a similar data set. More recently, Towers et al.^18^ estimated \begin{equation*}\small{ R_0}=1.2\end{equation*} (1.0,1.5) for Sierra Leone using a longer timeseries of case reports and a model with time-dependent reproduction numbers. And, the WHO Ebola Response Team^1^ presents an estimate of \begin{equation*}\small{ R_0}=2.02\end{equation*} (1.789-2.26) for Sierra Leone, which additionally makes use of new information about the incubation period and serial interval for the current epidemic. The analysis by Althaus modeled the natural history of infection by including a mean 5.3 day latent period before cases become infectious, whereas the analysis by Fisman et al. used a much longer latent period, but did not explicitly consider the lack of infectiousness during the latent period.

We conduct a phylodynamic^21^ analysis of 78 Ebola virus genetic sequences discussed in Gire et al.^16^. These data provide an independent source of information about epidemic growth rates and \begin{equation*}\small{R_0}\end{equation*} and may corroborate previous estimates based on case reporting data. To examine the sensitivity of \begin{equation*}\small{R_0}\end{equation*} estimates to the unknown latent period, we repeat our analysis with two values (5.3 and 12.7 days) which have been estimated from previous Ebola outbreaks.

Recently, Stadler et al.^34^ conducted a similar phylodynamic analysis of the same data. We conclude our analysis with a discussion of the primary differences in the analytic approach and findings of these two studies.

An advantage of phylodynamic analysis is that estimates are robust to incomplete sampling of cases, and the proportion of cases which are unreported does not enter directly into our model^24^ . Sequence data may also be informative about epidemiological parameters where standard surveillance data are unhelpful. Previous phylodynamic analyses have shown how sequence data can be highly informative about who infected whom^26^ and risk factors for transmission^25^. In addition to $R_0$ , we estimate parameters that describe heterogeneity in transmission rates between infected individuals. Fitting these models allows us to characterize superspreading as well as estimate the proportion of cases which do not yield secondary infections.

We also examine the virus genomes for evidence of natural selection, which can potentially bias phylodynamic analyses by violating assumptions of neutral evolution. Because the primary analysis of EBOV isolates 16 found a large number of non-synonymous mutations in whole length genomes, and because strong selective pressures can bias molecular clocks^10^^,^ 11, and violate the assumptions of the standard coalescent process^9^, we performed an exhaustive analysis of all genes in the EBOV genome for evidence of episodic diversifying natural selection using sensitive codon-substitution evolutionary models.

## Methods

**Data.** We conduct a secondary analysis of EBOV phylogenies presented by Gire et al.^16^ Samples were collected for whole-genome deep sequencing from 78 patients between 25 May and 20 June in Sierra Leone. In situations where multiple samples were available for a single patient, only the first sample was used in the phylogenetic analysis. Dates of common ancestry for all pairs of samples were estimated with using Bayesian relaxed clock methods 28 . Further details of the sequencing protocol and models used for phylogenetic analysis can be found in Gire et al. 16 This procedure yields a sample from the posterior distribution of dated phylogenies, from which we sampled 1,000 trees to make computation tractable.

For molecular selection analyses, we augmented the 2014 outbreak sequences with 17-37 (depending on the gene) additional isolates from previous EBOV outbreaks (1976-2007), which were also included in the original Bayesian relaxed clock analysis 16.

Cumulative numbers of cases reported by WHO were acquired from *https://github.com/cmrivers/ebola* on September 13, 2014.

**Models.** The starting point for the analysis is the SEIR model, which has previously been applied to EBOV outbreaks 29 ^,^ 30 and has recently been applied to the 2014 epidemic 19 .

The parameter β will be the transmission rate per infectious individual, \begin{equation*}\gamma_E\end{equation*} will be the rate that infected progress from the latent period to the infectious period, and $\gamma_I$ will be the rate that infectious cases are removed due to death and burial or by effective isolation and treatment. The ordinary differential equations for the deterministic SEIR model are:

SEIR equations. \begin{equation*} \begin{align}\%0A\frac{\mathrm{d}}{\mathrm{d} t} E \%26= \beta I S / N - \gamma_E \\\%0A  \frac{\mathrm{d}}{\mathrm{d} t} I \%26= \gamma_E E - \gamma_I I \%0A        \end{align}\end{equation*}

These equations describe the dynamics of the number $E$ exposed non-infectious individuals and the number of infectious individuals $I$ . We make the approximation that the majority of the population is in the susceptible category ( $S/N\approx 1$ ) for this and subsequent models, such that an equation for the dynamics of $S$ is not needed.

In order to estimate heterogeneity in transmission rates, we extend the SEIR model to include two infectious categories, $I_l$ and $I_h$ . When the latent period ends, a case progresses to the category \begin{equation*}I_h\end{equation*} with probability $p$ and category $I_l$ with probability $1-p$ . According to this model, transmissions only occur from the category $I_h$ . By changing the parameter $p$ , the variance in transmissions per infectious case can be made arbitrarily large. The deterministic equations are:

**ODE SEIIR equations.**
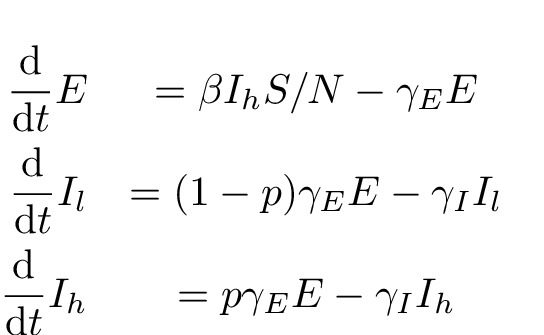

We will refer to this as the ODE SEIIR model.

A stochastic version of the SEIIR model was also fitted in order to account for any bias due to noisy dynamics during the early exponential growth phase of the epidemic. The equations for this model are given by the following stochastic differential equations:

**SDE SEIIR equations.**
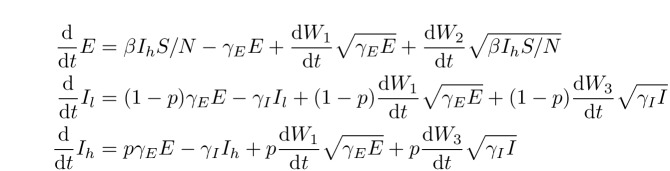

where $W_i(t)$ are independent Wiener (standard Brownian motion) processes, and $I=I_h+I_l$ . This system accounts for noise in incidence and deaths, but does not account for noise in the composition of $I$ into the $I_l$ and $I_h$ categories because of the difficulty of fitting such a system to data. Note that the stochastic terms in the equations for $I_l$ and $I_h$ are the same and multiplied by $1-p$ and $p$ respectively. We will refer to this model as the SDE SEIIR model. The euler method with a time step of one day was used to simulate trajectories from the SDEs.

**Statistical analysis.** The epidemiological models were fitted to EBOV phylogenies using the *rcolgem* package in R^22^^,^ 23, which computes the likelihood of epidemiological parameters given a phylogeny. When fitting the ODE and SDE SEIIR models, likelihoods were calculated for each phylogeny with the boundary condition that each sample is a superspreader with probability $p$ , which is estimated. Models were fitted using a Bayesian Markov chain within Metropolis (MCWM) algorithm^15^ , which integrates over the distribution of phylogenies previously estimated in Gire et al.^16^ . This algorithm was implemented by customizing the *mcmc* package in R. At each step $k$ of the MCWM algorithm, the likelihood of the set of trees $\{\mathcal{G_i}\}_{i=1}^n$ given a solution $\mathcal{M}_k$ of the epidemiological model is approximated by $$  \hat{\mathcal{L}}_k = \frac{1}{n} \sum_{i=1}^n P(\mathcal{G}_i | \mathcal{M}_k) $$ The $n$ genealogies used in the approximation are drawn uniformly at random from the distribution estimated by Gire et al. 16 If fitting a stochastic model, a double-marginalization is required over genealogies and simulations of the stochastic model. Such a Markov chain will sample the posterior distribution regardless of the choice of sample size \begin{equation*}   $n$   \end{equation*} , however the value used will influence the efficiency of the algorithm. We chose $n=4\times 16 = 64$ for ODE models and $5\times 64 = 320$ for SDE models to match the architecture of our high performance computing cluster.

In the SEIR model, two parameters were estimated: $R_0$ and the time $t_0$ when the epidemic was initiated in Sierra Leone. In the ODE SEIIR and SDE SEIIR model, the parameter $p$ was also estimated, which controls the proportion of cases in $I_h$ . A diffuse lognormal prior (mean 3.2, standard deviation 2.5) was used for $R_0$ , a uniform(0,1) prior was used for $p$ , and a normal prior for $t_0$ with mean April 23, and standard deviation of 6 days (based on the results by Althaus^19^).

We compared models using the approximate AICM method^32^. AICM is a summary statistic for the goodness of model fit for Bayesian analyses, and is analogous to the Akaike information criterion (AIC^8^) used for model selection in the maximum likelihood framework. AICM was calculated using Tracer1.6^14^ . At least two MCWM chains were sampled for each model and combined with 20% of samples removed for burn-in and effective sample sizes were computed to confirm adequate sample size.

The reproduction number was calculated using $R_0=\beta / \gamma_I$ for the SEIR model and $R_0=p \beta / \gamma_I$ for the SEIIR models. The coefficient of variation (CV) was computed under the assumption that if an individual is infectious, the number of transmissions has a geometric distribution with parameter \begin{equation*}\gamma_I / (\gamma_I + \beta)\end{equation*} . For a geometric distribution under the SEIR model, CV= $\sqrt{(R_0 + 1)/R_0}$ . According to the SEIIR model, an individual is infectious with probability \begin{equation*} $p$\end{equation*} , which yields CV = \begin{equation*}\sqrt{(R_0 (2/p - 1) + 1)/R_0}\end{equation*} .

**Selection analyses.** We extracted sequences spanning the complete lengths of the seven annotated genes of EBOV^16^ and fitted several evolutionary models to multiple sequence alignments (MSAs) using fixed maximum clade credibility (MCC) trees computed from previous BEAST runs. MSAs were easily obtained because of the lack of indel variation in EBOV. We used sensitive methods for detecting episodic diversifying selection at the level of individual sites (mixed effects model of evolution^5^), individual branches (branch-site random effects likelihood, BSREL^6^ ), and a modification of the BSREL to test for gene-wide selection operating on the (monophyletic) clade of 2014 EBOV sequences. Let $\omega$ denote the ratio of non-synonymous to synonymous substitution rates. Briefly, whereas the original BSREL method describes the evolutionary process by fitting a model with a mixture of three separate \begin{equation*}\omega\end{equation*} values for each tree branch, we partitioned the tree into the foreground (the 2014 Western Africa EBOV clade) and background (all other branches) segments, and fitted two 3-bin $\omega$ distributions jointly to all the branches in each partition. A likelihood ratio test of the unconstrained model versus the null model where all $\omega \leq 1$ (i.e. negative selection or neutral evolution) was used to establish significance for evidence of diversifying positive selection affecting a proportion of sites along a proportion of 2014 EBOV lineages. All analyses have been implemented and run using HyPhy v2.12 7.

## Results

Table 1 shows parameter estimates based on four epidemiological models. Estimates of $R_0$ based on the simple SEIR model are similar to those based on the ODE SEIIR model (posterior median $R_0=2.14,~ 2.10$ respectively), however the SEIR model does not provide an estimate of heterogeneity in transmission rates. The stochastic SEIIR model gives similar estimates to the deterministic SEIIR model, but wider credible intervals, and a slightly larger $R_0$ of 2.40 (95% HPD:1.54-3.87). These estimates are broadly consistent with the previously published estimates in Althaus 19 and by the WHO Response Team 1 which were based on WHO case reporting in Sierra Leone.

**Figure:**
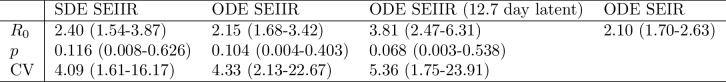
Table 1. Posterior median and 95% credible intervals based on four epidemiological models. Unless stated otherwise, each model assumed a mean 5.3 day latent period.

Estimates of $R_0$ are sensitive to the latent period which could not be estimated from the genetic data alone. Published estimates of the duration of the latent period based on earlier Ebola outbreaks are highly variable 31 , but we present results based on two values that have been used in recent modelling studies of the current epidemic in Western Africa. If the latent period is a mean of 12.7 days, the fitted ODE SEIIR model provides an estimate of $R_0=3.8$ (95% HPD:2.47-6.31).

**Cumulative number of symptomatic infections through time.**
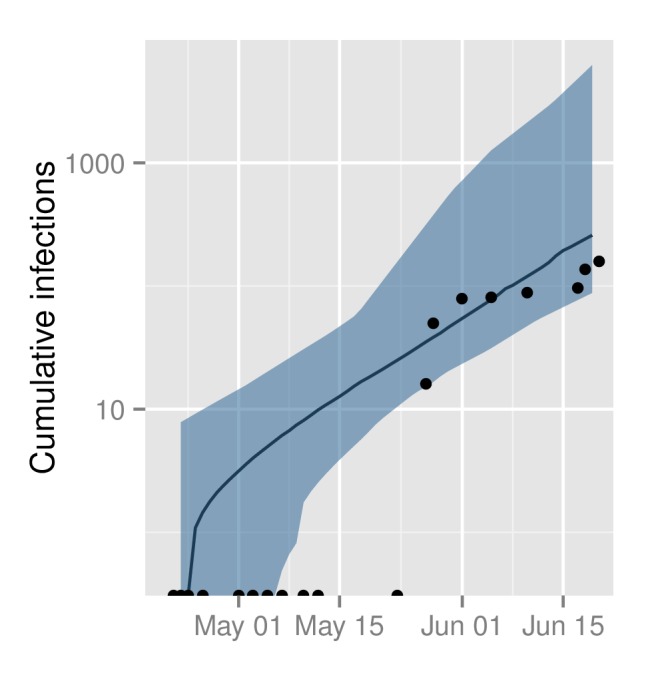
The estimated cumulative infections through time using the ODE SEIIR model. The shaded region represents the 95% HPD region and the line is the median. The points represent the cumulative number of infections (confirmed and probable) reported by WHO.

Figure 4 shows the estimated cumulative number of infections through time as predicted by the ODE SEIIR model with \begin{equation*}1/\gamma_E = 5.3\end{equation*} days. These estimates, while imprecise, are consistent with reported cases by WHO which were not used for model fitting. The slightly larger number of cases predicted by the model may in part reflect under-reporting of cases in the WHO data.

We find strong evidence for superspreading. The fitted SEIIR models which account for supserspreading have higher median posterior log likelihoods of -346.9 versus -380.1 for the SEIR model. The ODE SEIIR model is also superior to the SEIR model by the AICM criterion. For two distinct Markov chain samples, we find $\Delta$ AICM=-38.7 and -25.6 in favor of the ODE SEIIR model.

In all fits of the SEIIR model, the estimated proportion of cases in the high transmission rate category is less than 63% and posterior median estimates are approximately 10%. Figure 5 shows the EBOV phylogeny with maximum posterior probability. Branches are colored with the probability that the virus lineage inhabits a superspreading host. The superspreader lineage probabilities are based on the median posterior parameter estimates with the ODE SEIIR model. When a lineage occupies a superspreading host (shaded red), it is much more likely to undergo a coalescent event, that is, to have common ancestry with other sampled lineages. This process yields phylogenies with very imbalanced topologies. It also introduces correlation between the lengths of neighboring ancestral and daughter branches, as a lineage in a superspreading host is likely to undergo several coalescent events in short succession.

**EBOV phylogeny.**
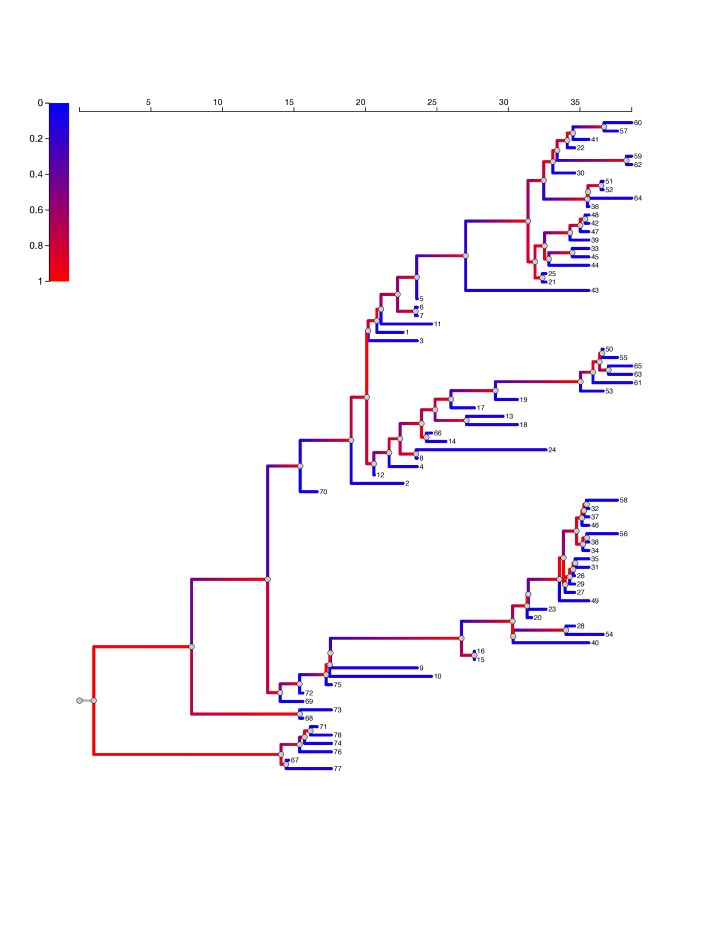
Ebola virus phylogeny based on samples from 78 patients in Sierra Leone showing superspreading. The color of branches represents the estimated probability that the virus lineage inhabits a superspreading host. This phylogeny had the maximum sampled posterior probability in the phylogenetic analysis in Gire et al.^16^ Estimates are based on the median posterior parameter estimates with the ODE SEIIR model.

**Selection analyses. ** Table S1 provides an overview of codon-based selection analyses of the seven EBOV protein coding regions. There is a notable variation in nucleotide level diversity across genes (total tree length), with the glycoprotein (GP) showing the highest diversity. About 0.5% of branch-site combinations in the long RNA polymerase (L) gene appear to be under strong diversifying selection ( $\omega>100$ ) in the 2014 clade, whereas the entirety of sequence evolution in the GP gene is comprised of non-synonymous changes ( $\omega > 1$ at 100% of branch-site combinations). The remaining genes do not contain significant positive selection signal.

When we asked which individual sites showed evidence of episodic diversification (using the MEME^5^ method with p-value < 0.95), sites 388 and 389 in the heavily glycosylated mucin-like domain of GP, and sites 1396, 1492, 1722 in L were identified.

## Discussion

Phylodynamic analysis of EBOV sequences provides a new perspective on $R_0$ and epidemic growth rates that is independent of previous analyses based on WHO case reports. Previous analyses have reached divergent conclusions about $R_0$ in Sierra Leone, and our estimates are consistent with previous analyses in Althaus^19^ and by the WHO Ebola Response Team^1^ if assuming a short latent infection period, but slightly larger if assuming a longer latent infection period. Our results are sensitive to the early evolutionary history of the Sierra Leone EBOV, much of which occurred before the first WHO case reports. Thus, the discrepancy of our results with the studies by Towers et al.^18^, who reported smaller values of $R_0$ , may be due the decrease in epidemic growth rates observed in July and August. Estimates of $R_0$ are very sensitive to the unknown duration of the latent period which can not be estimated from genetic data alone. Recently, the WHO Ebola Response Team published its first estimates of the incubation period and serial intervals for the 2014 epidemic^1^, and found a mean incubation period of 11.4 days. This suggests that $R_0$ is closer to the upper bound of our range of estimates (2.10-3.85). Stadler et al.^34^ recently estimated \begin{equation*}R_0\end{equation*} =2.18 (95% HPD 1.24-3.55) using the same genetic data used in our analysis. These results are close to our findings if comparing similar models (SEIR and BDEI) and similar incubation periods. The credible intervals in the analysis by Stadler et al.^34^ are much wider because a diffuse prior was used for the latent period, whereas we tested the sensitivity to this parameter by repeating the analysis with the latent period fixed at different values.

While we find that it is not possible to estimate the latent period from genetic data alone, Stadler et al.^34^ have conducted a phylodynamic analysis of the same EBOV data and estimated a mean incubation period (assumed equivalent to the latent period) of 4.92 days (95% HPD 2.11-23.20). Stadler et al.'s inference of incubation periods was made possible by using additional information, namely the times of genetic sequence sample collection, which were assumed to be collected at a constant per-capita rate. By calibrating the exponential growth rates of the epidemic to match the rate of sample collection, other parameters are rendered identifiable. Incorporating a model of the sampling process into phylodynamic inference can greatly increase statistical power^24^, however it can also bias estimates if the sampling process is misspecified. We do not find evidence that the sampling rate was constant as required by the analysis in Stadler at al.^34^, but rather that it increased steadily over the sample collection period. By comparing the cumulative number of infections reported by WHO to the cumulative number of samples collected, we find that the sampling rate varied from 20% early in the epidemic to 70% near the end of the sample collection period. An alternative to using times of sample collection to calibrate growth rates would be to use the WHO case reports. Unfortunately, there is very little overlap between the time-stamped EBOV phylogenies and WHO case reports because all samples were collected during the early portion of the epidemic.

In contrast to the analysis by Stadler et al.^34^ , we find statistically significant support for a model which features superpreading (heterogeneous transmission rates). These divergent findings may be due to differences in the population genetic models used (coalescent and birth-death-sampling). The discrepancy may also be due to a different parameterisation of superspreading. The analysis by Stadler et al. required two additional parameters to describe superspreading. We chose a model of superspreading which required only one additional parameter, thereby increasing discriminatory power at the expense of some realism. The quantitative estimates of the CV of the reproduction number may be biased upwards by unrealistic distributional assumptions in our model. It is unlikely that transmission heterogeneity is well described by a mixture of only two transmission rates.

It is possible to characterize superspreading patterns from virus phylogenies because the variance in transmissions per case alters the genetic relatedness of a random sample of EBOV sequences^27^. The EBOV phylogenies are highly imbalanced, and neighbouring branch lengths are highly correlated. We hypothesize that these features are a consequence of high variance in transmission rates, and we have proposed an epidemiological population genetic model that reproduces these features. Our epidemiological model of superspreading lacks some realism, however our parameterisation of the transmission process allows us to easily estimate variance in transmission rates.

High variance in transmission rates may hamper contact tracing efforts, since a single missed contact may trigger a sizeable outbreak. Epidemics which feature a highly skewed distribution of transmissions per infected individual differ substantially from epidemics where the number of transmissions cluster around $R_0$ 3 ^,^ 2 . In epidemics with many superspreading events, the probability of epidemic extinction is greater, and the probability that a single introduction into a susceptible population will trigger an epidemic is also lower. But, when outbreaks do occur, they are more explosive and contact tracing may be more difficult. Furthermore, intervention strategies that are targeted towards individuals with higher transmission risk are likely to be more effective in epidemics with superspreading events. We estimate that a small proportion of infected cases are responsible for a majority of transmissions and a large proportion of infections yield no transmissions.

Our molecular selection analyses suggest that episodic diversifying selection may be operating on L and GP genes. When analysing recent viral isolates, much of the selection signal could be driven by overall maladaptive substitutions along terminal branches due to intra-host evolution 4. When additional isolates become available, some of the techniques for filtering out such substitutions (e.g. analysing only internal branches 4 ) may prove fruitful. The functional importance of sites subject to such forces remains to be elucidated.

Many factors contribute to the uncertainty of our findings, including uncertainty in the EBOV phylogenies, dates of common ancestry, and inherent noisiness of the epidemic process during the early period of exponential growth. Our estimates are based on a relatively small sample of EBOV sequences, and much greater precision could be achieved if a larger proportion of cases are sequenced over a longer period of time. Genetic sequence data are only available for the very early portion of the epidemic in Sierra Leone. Estimates may differ in other countries and settings, as well as through time as intervention efforts are scaled up and the population adapts to the growing epidemic. Phylodynamic methods are robust to variable and incomplete sampling of cases, so that virus sequences may be a useful supplement to epidemic surveillance if a growing proportion of cases are not reported to health systems.

## Competing Interests

The authors have declared that no competing interests exist.

## Figures and Tables

**Figure:**
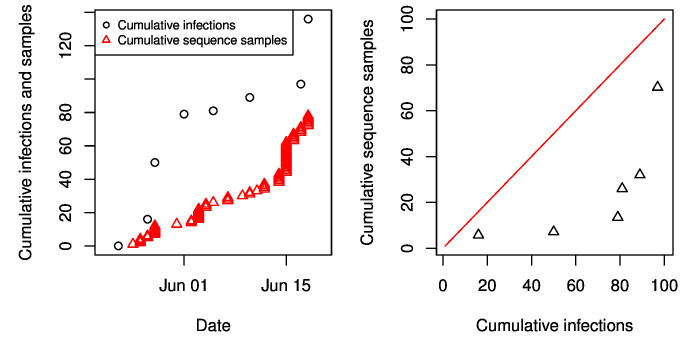
Fig S1: Sampling through time. Left: The cumulative sequence samples as a function of time are compared to cumulative confirmed and probable cases reported by WHO. Right: The WHO cumulative infections versus the interpolated cumulative number of sequence samples. If the sampling rate was constant, these points would follow a linear trend.

## Appendix 1

### Supporting Tables and Figures

**Table S1: Selection analysis.** Selection analysis results on EBOV genes; N, number of sequence; L, number of codons; T, total tree length, expected substitutions/nucleotide site. $\omega^{2014}$ is the inferred distribution of the ratio of non-synonymous to synonymous substitution rates under the modified branch-site REL model (see text) on the clade comprising 2014 outbreak isolates (weight of each category is indicated in the parentheses); for the two genes with significant p-values, we also indicate approximate 95% confidence intervals (likelihood profile) for the ω categories with estimates over 1 in square brackets; $\omega^{other}$ is the corresponding distribution for the remainder of the tree (pre-2014); $p$ is the p-value for the likelihood ratio test for evidence of episodic diversifying selection anywhere in the 2014 clade.
